# Understanding the role of discriminative instruments in HRQoL research: can Ferguson's Delta help?

**DOI:** 10.1186/1477-7525-6-82

**Published:** 2008-10-16

**Authors:** Kathleen W Wyrwich

**Affiliations:** 1 Senior Research Scientist, United BioSource Corporation, Bethesda, MD, USA

## Abstract

A critique of Hankins, M: How discriminating are discriminative instruments?" *Health and Quality of Life Outcomes *2008, **6**:36

## 

As a PhD student in the 1990s, one of my favorite places on campus was the Lewis Annex to our university's library. It is an underground shelf space with limited accessibility, very low ceilings and dim lighting. However, hidden in the damp Lewis Annex were bound volumes of *Psychometrika *dating back to the 1930s. Alone with no other library patrons near, I could spread open several articles at once on the floor and follow the thoughts of past psychometric sages late into the night with great interest and content, with complete abandonment of time and my real-world responsibilities outside of those yellow-paged tomes and narrow library aisles.

As I read Matthew Hankins' paper on the use of Ferguson's Delta [1] as an index of discriminate validity, the smell and fond memories of the Lewis Annex swept over me like a soft cloud. However, not only did this paper trigger a reflection on the psychometric masters of the mid 20^th ^century, it caused me to contemplate the debt of gratitude I and other health outcomes researchers have to the late 20^th ^and 21st century health researchers at McMaster University who have taken the knowledge written and stored in the bowels of the Lewis Annex to applications featured in the center of all brightly-lit health science libraries, and, indeed, informing and changing the practice of health care.

Unfortunately, in order to make a case for the needed usefulness of Ferguson's Delta, Hankins has not embraced the purpose or details of the Kirshner and Guyatt's 1985 taxonomy paper, or the relevant work by these authors beyond 1992. The bibliography in Hankins' paper leads me to fear that he has missed the rich Evidence-Based Medicine series published in *JAMA *and other insightful theoretical and clinical applications for the measurement and evaluation of HRQOL measures that have emerged over the past 3+ decades among McMaster heath science researchers.

The stated intent of 1985 taxonomy was to simplify the chaos in the health status measurement literature with three classifications of health instruments by their purposes: predictive, discriminative and evaluative [2]. In 1995, Guyatt further addressed and elucidated the properties of discriminative instruments, which are: 1) reliability; 2) correlations between measures as a point in time consistent with theoretical predictions; and 3) differences between subjects at a point in time can be interpreted as trivial, small, moderate or large [3].

When considering the reliability of a discriminate HRQOL measure, the taxonomy papers have repeatedly stressed this concept in terms of signal and noise where "reliable instruments will generally demonstrate that stable subjects show more or less the same results on repeated administrations." p. 1188 [3] In the HRQOL literature, this is commonly referred to as test-retest reliability. Yet in none of the Hankins examples is this type of reliability calculated or reported. The first example uses an intraclass correlation between *two different *measures as the estimate of reliability, while the second and third examples use Cronbach's alpha to judge reliability. Both of these methods for estimating reliability are inappropriate for assessing this property of discriminative measures, as described by Kirshner and Guyatt [4].

The second property of a discriminative instrument is a cross sectional relationship with a theoretical criterion or prediction, and again, in none of the examples offered by Hankins do we see this very important property demonstrated. We are told in Example 1 that the two measures being compared are "equally valid," but no explanation is given to allow the reader to know the basis of this validity assessment. Moreover, no results on the relationship of the reported measures or items in Example 2 and 3 are provided.

Likewise, the third and perhaps most important property that the McMaster group has endorsed for discriminative instruments is interpretability so that small but important cross sectional differences between subjects are distinguishable. This property is not addressed in the Hankins' paper, and there is no explanation for how the use of Ferguson's Delta would enhance interpretability.

Although the use of Ferguson's Delta may someday improve our understanding of discriminative measures and their development, the examples supplied in this paper do not allow us to currently make this judgment. Hankins' prior published work applying Ferguson's Delta to identify the discrimination of dichotomous vs. 4-point Likert scaled GHQ-12 items gave results that are well-expected; Likert response items (if chosen correctly) are more discriminating between individuals than dichotomous items [1]. It is important to note that Guyatt, Kirsner and Jaeschke expressed that the "evidence for the success [of their taxonomy] would be the students' ability to manipulate concepts and to produce higher quality research from a sound conceptual basis." p. 1353 [5] Hence, we look forward to seeing relevant demonstration of the usefulness of this novel psychometric method in HRQOL research that fully encompasses the intent of the taxonomy, integrates the relevant properties described above, and reflects the McMaster authors' goal for evidence of its of success.

## Competing interests

The author declares that she has no competing interests.
